# Immune checkpoint changes correlate with the progression and prognosis of amyotrophic lateral sclerosis

**DOI:** 10.1080/07853890.2025.2540023

**Published:** 2025-08-03

**Authors:** Sheng Chen, Chunzuan Xu, Changyun Liu, Jiaqi Li, Shupei Ke, Yingqian Lu, Yali Huang, Jialin Chen, Feifei Lin, Huapin Huang, Zhangyu Zou

**Affiliations:** a Department of Neurology, Fujian Medical University Union Hospital, Fuzhou, China; b Institute of Clinical Neurology, Fujian Medical University, Fuzhou, China; c Clinical Research Center for Precision Diagnosis and Treatment of Neurological Diseases of Fujian Province, Fuzhou, China

**Keywords:** Amyotrophic lateral sclerosis, immune checkpoints, PD-1, prognosis

## Abstract

**Background:**

Amyotrophic lateral sclerosis (ALS) urgently requires robust biomarkers for early diagnosis and prognostic stratification. This study aims to investigate the diagnostic and prognostic potential of membrane-bound and soluble immune checkpoint molecules in ALS pathogenesis.

**Methods:**

In the present study at Fujian Medical Union Hospital, 72 participants (46 ALS and 26 healthy controls [HC]) underwent flow cytometry analysis of PD-1 expression in CD4^+^ T cells and its subsets. A second cohort (*n* = 93, 44 ALS, 30 HC and 19 ALS mimics [Mimics]) was evaluated using Luminex technology for 14 serum immune checkpoint molecules. A single-molecule array was used to screen the neurofilament light chain (NFL) in serum.

**Results:**

Flow cytometry revealed elevated PD1 expression in CD4^+^ T cells, particularly in Th9 and Th17 subsets (*p* < 0.05). ALS patients exhibiting a greater percentage of PD-1 in CD4^+^ T cells showed accelerated functional decline. Serum analyses identified four elevated soluble checkpoints in ALS versus both HCs and Mimics (sPD-1/sBTLA/sCTLA-4/sCD27, *p* < 0.05), with sCD28/TIM-3 showing higher in ALS than in Mimics, and sGITR/sCD137/sIDO/sCD80/sLAG3/sPD-L2 elevating in ALS compared to HCs. Soluble TIM-3 correlated inversely with ALSFRS-R, while sPD-L1 demonstrated dual associations: negative with ALSFRS-R and positive with NFL (all *p* < 0.05).

**Conclusions:**

Our research demonstrated a considerable increase in membrane-bound and soluble PD-1 in ALS patients, correlating with disease progression and worse prognosis. Furthermore, we explored 13 other immune checkpoint molecules. Collectively, these molecules may be implicated in peripheral immune mechanisms underlying ALS pathogenesis. While baseline PD-1 levels show some association with prognosis, their elevation potentially indicates an unfavorable course.

## Background

As a progressive, lethal neurodegenerative disorder, amyotrophic lateral sclerosis (ALS) leads to muscle atrophy, weakness, dysarthria, and dysphagia. The exact pathogenesis of ALS remains unclear, making diagnosis particularly challenging, especially in the early stage [1]. Studies involving animal models and patients of ALS have confirmed that dysfunction in the immune system serves a crucial role in the occurrence and progression of ALS [2,3]. Our previous research indicated that a higher proportion of CD4^+^EOMES^+^ T-cell subset is associated with disease severity and poor prognosis in ALS [4]. We also revealed changes in cytokine profiles among ALS patients [5].

The immune checkpoint pathway is essential for maintaining homeostasis in the immune system. Programmed death-1(PD-1), one of the most well-known immune checkpoint molecules, is established as an inhibitory receptor. Programmed death ligand-1 (PD-L1) and PD-L2 interact with PD-1 to form the PD-1 pathway [6,7], which negatively impacts T cell responses rather than directly regulating apoptosis or programmed cell death. Signaling pathways activated by B and T cell antigen receptors lead to the expression of PD-1 [8], which in turn inhibits immune responses [9,10]. As an inhibitory receptor, PD-1 actively suppresses the function of exhausted T cells [11]. In chronic infections and cancers, where persistent antigenic stimulation causes T-cell depletion and a gradual decline in effector function, monoclonal antibodies that inhibit the PD-1 pathway can partially restore these T-cells and have been employed in the treatment of various cancers [12,13].

Given the immune abnormalities observed in ALS, we wondered whether PD-1 and other immune checkpoint molecules undergo alterations in this condition. Several prior studies have hinted at the shifts in PD-1 levels in ALS as well [14]. Research on superoxide dismutase 1 (SOD1) transgenic mice has unveiled a marked elevation in effector T cells in the mouse spleen, along with substantial infiltration of a cluster of CD4^+^T cells expressing both PD-1 and activation markers into the spinal cord [15]. Additionally, studies involving ALS patients have shown a notable elevation in serum levels of PD-1 and PD-L2, indicating the presence of a peripheral pro-inflammatory response [14]. However, the levels of membrane-bound PD-1 have not been assessed in these studies, nor have the serum levels of other checkpoint molecules been evaluated. Therefore, we aimed to explore PD-1 expression on CD4^+^T cells in peripheral blood and the presence of immune checkpoint molecules, including PD-1, in the serum of ALS patients. Our goal is to explore these molecules in ALS and identify potential novel targets for therapy.

## Methods

### Participants and longitudinal follow-up

This study employed a two-phase recruitment strategy at Fujian Medical University Union Hospital. The flow cytometry cohort comprised 72 participants (46 ALS patients and 26 healthy controls [HC]), while the Luminex cohort included 93 subjects: 44 ALS patients from the flow cytometry cohort, 30 new HC, and 19 ALS mimics (Mimic). The ALS mimic group encompassed: (1) immune-related peripheral neuropathy (PN; *n* = 9) including 5 patients with chronic inflammatory demyelinating polyneuropathy (CIDP), 1 patient with IgG4-related disease, 1 patient with anti–myelin-associated glycoprotein (anti-MAG) neuropathy, 1 patient with Guillain Barre Syndrome (GBS), and 1 patient with lumbosacral radiculitis. (2) structural pathologies (cervical spondylosis, *n* = 3; single nerve injury, *n* = 2); (3) rare conditions (paraproteinemia-related PN, *n* = 2; immune-mediated necrotizing myopathy (IMNM), *n* = 1; post-polio syndrome, *n* = 1; spastic paraplegia, *n* = 1). All ALS patients were diagnosed by at least two experienced neurologists in accordance with the revised EI Escorial criteria [16]. The inclusion criteria encompassed a diagnosis of definite, clinically probable, and clinically probable-laboratory-supported ALS. Exclusion criteria for ALS, Mimics, and HCs comprised: (1) family history of ALS; (2) acute or chronic infections (confirmed by medical history review, clinical symptom assessment, and laboratory testing); (3) previous diagnosis of other neurodegenerative diseases; (4) administration of immunomodulatory drugs (including intravenous immunoglobulin) at the moment of blood sample collection. The exclusion criteria for ALS and HCs also comprised a history of autoimmune disorders. Participants underwent longitudinal monitoring at 12-month intervals for 12 months. During this period, we tracked ALS Functional Rating Scale-Revised (ALSFRS-R) scores, calculated the ALSFRS-R progression rate using formula [(48-ALSFRS-R Score)/Disease Duration (months)], and monitoring primary endpoint events, such as prolonged use of a non-invasive ventilator (more than 22 h per day), invasive mechanical ventilation, or death. We received approval from Fujian Medical University Union Hospital’s ethics committee (2019GZR032). Written informed consent was obtained from each participant.

### Blood sampling and flow cytometry

Peripheral blood samples with EDTA anticoagulated were collected and transferred to the laboratory for flow cytometry analysis within 2 h. For surface staining, a 100ul aliquot of peripheral blood was used, following the Human Immunophenotyping Consortium’s standardized phenotyping protocols [17]. We utilized a detailed panel of antibodies, including anti-CD3, anti-CD4, anti-CXCR3, anti-CXCR5, anti-CCR4, anti-CCR6, anti-PD1 [17–19]. Following staining, red blood cell lysis was performed using lysing buffer (Vazyme). Flow cytometry was conducted using a BD FACSCelesta^TM^ Flow Cytometer (BD Biosciences), and the Treestar FlowJo v10.6.2 (FlowJo, LLC) was used for data analysis.

### Gating strategies

The antibodies used for flow cytometry are detailed in Supplement Table 1, and gating strategies employed in this study are presented in Supplement Figure 1 (Table S1 and Figure S1). Briefly, the proportions of CD3^+^CD4^+^ T cells, as well as Th1, Th2, Th9, Th17, and Tfh were determined based on the expression of CD3, CD4, CXCR3, CXCR5, CCR4, and CCR6, according to previous reports [17–19]. Additionally, PD-1 levels in T cells and their subsets were calculated as well.

### Serum checkpoint markers and NFL quantification

Blood samples was collected in Vacutainer tubes (with gel clot activator) and delivered to the laboratory within 2 h. Serum was separated and stored in liquid nitrogen for further research. The Human Immuno-Oncology Checkpoint Marker Panel (14 plex) (EPX14A-15803-901, Invitrogen) was used to measure the levels of 14 human checkpoint markers in serum, including serum B and T lymphocyte attenuator (sBTLA), serum Indoleamine-2,3-Dioxygenase (sIDO), serum lymphocyte activation gene-3 (sLAG-3), serum T-cell immunoglobulin and mucin-domain containing-3 (sTIM-3), soluble PD-1 (sPD-1), sPD-L1, sPD-L2, serum cytotoxic T lymphocyte-associated antigen-4 (sCTLA-4/sCD152), sCD80, serum glucocorticoid-induced tumor necrosis factor receptor (sGITR), serum herpesvirus entry mediator (sHVEM), sCD27, sCD28, sCD137/4-1BB. The experiment was conducted on the Luminex 200 system according to the manufacturer’s instructions. Briefly, serum samples were thawed on ice. Standards, quality controls, and samples (25 µL/well) were incubated with pre-mixed magnetic capture beads for 2 h at room temperature with shaking. After two washes, detection antibodies (25 µL/well) were added and incubated for 1 h, followed by streptavidin-PE incubation. After washing, fluorescent signals from all samples are detected in Luminex instrument, and data are analyzed using manufacturer provided software. Additionally, the NFL assay kits (103186, Quanterix) were utilized to measure the level of NFL, following the manufacturer’s guidelines, on the ultrasensitive single-molecule array (SIMOA) platform. All calibrations and blank controls were performed in duplicates, and the grouping of samples was kept confidential from the operator.

### Statistical analyses

R version 4.5.0 was utilized to conduct statistical analyses in this study. The data underwent a normality test in R. Normally distributed data were presented as mean [standard deviation (SD)] and analyzed using the *t* test. Non-normally distributed data were displayed as median [lower quartile, upper quartile] and assessed using the Wilcoxon test. For comparisons among three groups with non-normally distributed data, pairwise analyses were performed using the Wilcoxon test with Bonferroni correction. Spearman correlation analysis was employed to calculate correlations. Kaplan–Meier analysis was used for survival analysis. Statistical significance was set at *p* < 0.05.

## Results

### Demographics

Table 1 summarizes the demographic and clinical characteristics of the flow cytometry and Luminex cohorts. The majority of the participants in our cohort had a documented history of riluzole use, with its utilization rate specified in Table 1. No patients received edaravone treatment. No significant differences were observed in demographic variables between ALS patients and either HCs or ALS mimics.

**Table 1.** Demographic and clinical characteristics of patients.

	Cohort of flow cytometry			Cohort of Luminex				
	ALS(*n* = 46)	HC(*n* = 26)	^†^ *p*	ALS(*n* = 44)	HC(*n* = 30)	^†^ *p*	Mimic(*n* = 19)	*p
Sex, male, no. (%)	25 (54.35)	13 (50.00)	0.729	24 (54.55)	16 (53.33)	0.924	13 (68.42)	0.305
Age (mean ± SD, years)	55.98 ± 9.09	52.31 ± 12.64	0.200	56.39 ± 9.06	47.4 ± 4.49	0.172	51.21 ± 15.64	0.191
Disease duration (Median [IQR] months)	11.00 [6.00**–**20.00]	–		11.00 [6.00–19.25]	–		–	
Site of onset, no. (%)								
Bulbar	16 (34.78)	–		16 (36.36)	–		–	
Limb	30 (65.22)	–		28 (63.64)	–		–	
ALSFRS–R (Median [IQR])	40.50 [38.25–42.75]	–		41.00 [39.00–43.00]	–		–	
ALSFRS–R/month (Median [IQR])	0.79 [0.34–1.09]	–		0.79 [0.33–1.13]	–		–	
The usage rate of riluzole (%)	82.61	–		81.82	–		–	
King’s staging, no. (%)								
2 A	10 (21.74)	–		9 (20.45)	–		–	
2B	23 (50.00)	–		23 (52.27)	–		–	
3	11 (23.91)	–		10 (22.73)	–		–	
4	2 (4.35)	–		2 (4.55)	–		–	
5	0 (0.00)	–		0 (0.00)	–		–	
ALS-MiToS staging, no. (%)								
0	40 (86.96)	–		40 (90.91)	–		–	
1	4 (8.70)	–		2 (4.55)	–		–	
2	1 (2.17)	–		1 (2.27)	–		–	
3	1 (2.17)	–		1 (2.27)	–		–	
4	0 (0.00)	–		0 (0.00)	–		–	
5	0 (0.00)	–		0 (0.00)	–		–	

ALS, amyotrophic lateral sclerosis; HC, healthy control; mimic, patients with ALS mimic syndrome; SD, standard deviation; IQR, interquartile range; ALSFRS–R, amyotrophic lateral sclerosis; ALS–MiToS, ALS–Milano Torino functional staging.

^†^*p* values for ALS vs. HC comparison.

*p values for ALS vs. mimic comparation.

### Comparison of PD-1 expression in T cells between ALS and HCs using flow cytometry

**Figure 1.**
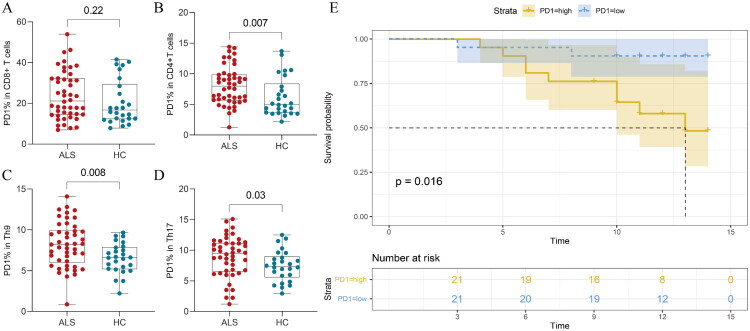
The levels of PD1 in T cells and Th subsets by flow cytometry and the survival curve in patients with ALS and HC. (A) No significant difference in the levels of PD1 in CD8^+^T cells between ALS patients and HC. (B) The levels of PD1 in CD4^+^T cells were significantly elevated in ALS patients. (C, D) Increased PD1 frequency in Th9 (C) and Th17 (D) subsets of ALS patients. (E) Survival analyses indicated that ALS patients with higher levels of CD4 + PD+T cells have a poor prognosis.

Flow cytometric analysis of PD-1 expression in peripheral blood T-cell subsets revealed distinct patterns in ALS patients versus HC (Table 2). While no significant difference was observed in the levels of PD-1 in CD8^+^T cells between groups (Figure 1A), ALS patients exhibited elevated proportions of PD-1 in CD4^+^T cells compared to HCs (8.36 [5.63–9.93] vs. 5.15 [3.83–7.09], *p* = 0.008, Figure 1B; Table 2). Additionally, the proportions of PD-1 in Th9 and Th17 subsets were higher in ALS patients than HCs (8.03 [5.77–9.83] vs. 6.66 [5.53–7.94], *p* = 0.009, and 9.18 [6.25–11.10] vs. 7.80 [6.60–9.37], *p* = 0.034, Figure 1C,D; Table 2). Notably, PD-1 expression levels showed no significant correlation with clinical parameters, including ALSFRS-R score, ALSFRS-R progression rate (ALSFRS-R/month), or NFL levels.

**Table 2.** The results of PD1 detected by flow cytometry.

	ALS (Median [IQR])	HC (Median [IQR])	*p*
Cohort of flow cytometry			
CD8^+^T (% in CD3^+^T)	38.85 [33.28–46.48]	34.90 [31.18–46.08]	0.333
CD4^+^T (% in CD3^+^T)	59.65 [50.58–66.73]	65.10 [55.70–68.83]	0.330
Th1 (% in CD4^+^T)	14.40 [11.50–16.58]	11.80 [9.67–14.25]	0.302
Th2 (% in CD4^+^T)	5.98 [4.46–9.70]	5.31 [4.15–7.22]	0.376
Th9 (% in CD4^+^T)	19.10 [15.35–27.25]	21.40 [12.65–23.80]	0.345
Th17 (% in CD4^+^T)	12.85 [9.39–17.60]	13.25 [6.91–16.53]	0.409
Th17.1 (% in CD4^+^T)	7.68 [5.44–9.51]	6.70 [5.71–8.24]	0.648
Tfh (% in CD4^+^T)	3.93 [2.55–5.36]	3.65 [2.18–5.56]	0.432
PD1 (% in CD8^+^T)	21.20 [14.59–32.07]	16.71 [12.51–27.52]	0.217
PD1 (% in CD4^+^T)	8.36 [5.63–9.93]	5.15 [3.83–7.09]	0.008
PD1 in Th1	8.79 [6.37–10.53]	7.23 [5.16–10.55]	0.118
PD1 in Th2	7.65 [5.22–9.83]	6.62 [5.22–9.86]	0.148
PD1 in Th9	8.03 [5.77–9.83]	6.66 [5.53–7.94]	0.009
PD1 in Th17	9.18 [6.25–11.10]	7.80 [6.60–9.37]	0.034
PD1 in Th17.1	6.61 [4.68–9.26]	6.05 [4.51–7.57]	0.086
PD1 in Tfh	10.07 [6.98–12.43]	9.44 [8.34–12.05]	0.774

ALS, amyotrophic lateral sclerosis; HC, healthy control; IQR, interquartile range; Th, helper T cell; Tfh, follicular helper T cell; PD1, programmed cell death protein 1.

To evaluate the prognostic relevance of PD-1 expression, we conducted a 12-month longitudinal follow-up. Among the 46 initially enrolled ALS patients, 42 patients completed follow-up (4 lost to attrition), with 11 reaching predefined endpoint events. Using the median proportion of CD4^+^PD-1^+^ T cells (8%) as the stratification threshold, Kaplan–Meier survival analysis demonstrated significantly accelerated endpoint progression in the high PD-1 group versus the low PD-1 group (*p* = 0.016, Figure 1E). In contrast, no significant differences in survival were observed between high and low PD-1 groups (using median expression levels as the threshold; CD8^+^: 21.20%; Th9: 8.21%; Th17: 9.47%) for CD8+ T cells, Th9 cells, or Th17 cells (*p* > 0.05, Figure S2).

### Quantitative profiling of circulating immune checkpoint proteins in ALS patients versus controls: a Luminex Technology-based multiplex immunoassay study

**Figure 2.**
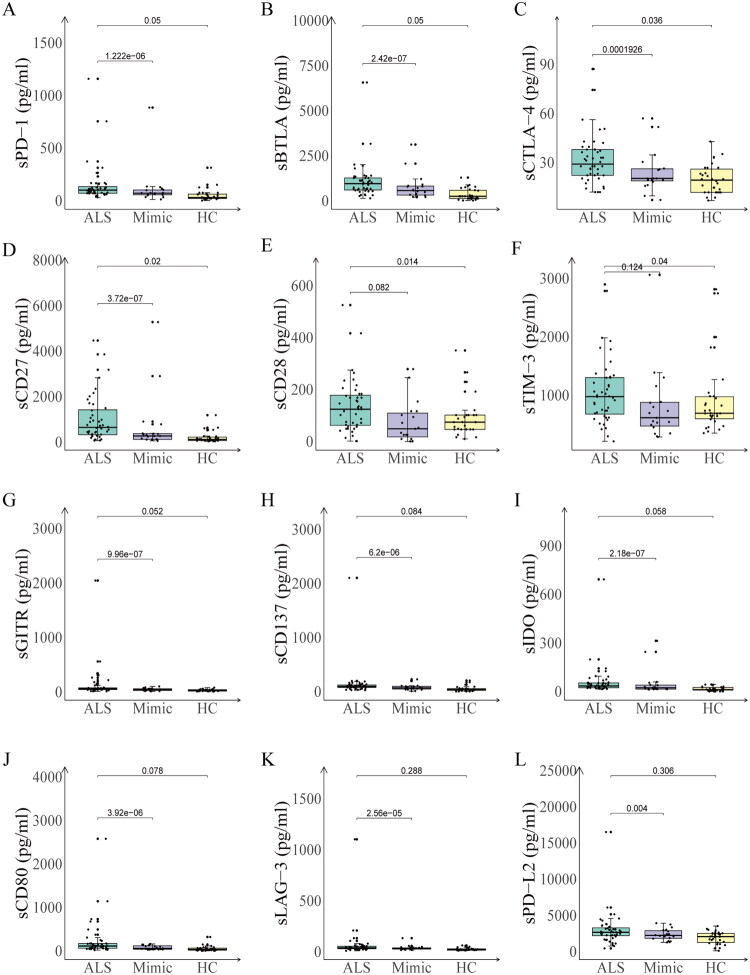
The levels of serum checkpoint markers tested by luminex in patients with ALS, HC, and mimic. (A–D) Serum levels of sPD-1, sBTLA, s-CTLA-4, and sCD27 were significantly elevated in ALS patients compared to both HC and mimic. (E–L) sCD28 and sTIM-3 was higher in ALS patients than in mimic, while sGITR, sCD137, sIDO, sLAG3, sPD-L2 and sCD80 were elevated in ALS patients compared to HC.

**Figure 3.**
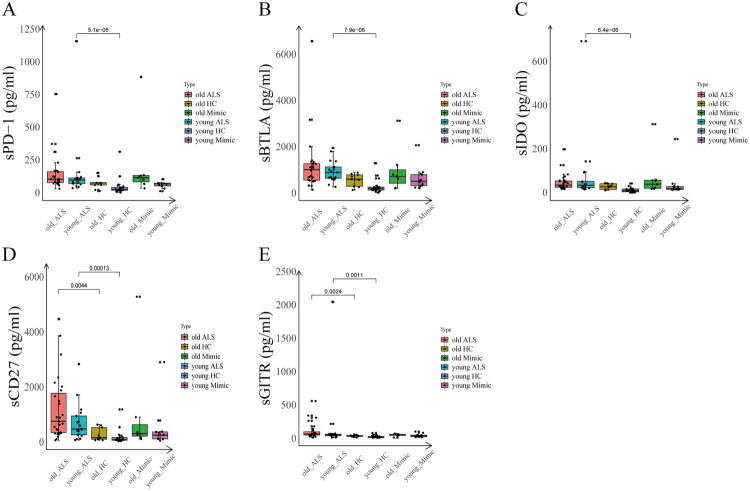
Serum levels of immune checkpoint markers tested by luminex in patients with ALS, HC, and mimic, stratified by medium age (53 years old). (A–C) The levels of sPD-1, sBTLA, and sIDO were significantly elevated in younger ALS vs younger HCs. (D–E) The sCD27 and sGITR levels increased in older ALS patients compared to older HCs, and in younger ALS patients relative to younger HC.

To delineate the systemic dysregulation of immune checkpoint networks in ALS pathogenesis, we employed a Luminex multiplex immunoassay platform to quantify 14 soluble immune mediators in three well-characterized cohorts: ALS patients(*n* = 44), age-matched HC (*n* = 30), and Mimics (*n* = 19) (Table 3). Notably, four analytes (sPD-1, sBTLA, sCTLA-4/sCD152, sCD27) demonstrated concurrent elevation in ALS patients versus both HC and Mimics (all *p* < 0.05, Figure 2A–D). Eight biomarkers exhibited differential specificity: sCD28, and sTIM-3 were higher in ALS patients than in Mimic, while sGITR, sCD137, sIDO, sLAG3, sPD-L2 and sCD80 were preferentially upregulated relative to HC (all *p* < 0.05, Figure 2E–L). When comparing specific diseases among Mimics, the levels of sPD-1, sBTLA, sCTLA-4/sCD152, sCD27, sCD28, sTIM-3, sGITR, sCD137, sIDO, sCD80, sLAG-3, and sPD-L2 were found to be comparable to those in some autoimmune diseases, such as CIDP and GBS (Figure S3A–L). Age-stratified analyses revealed multi-layered dysregulation patterns of soluble immune checkpoints (Figure 3). The levels of sPD-1, sBTLA, and sIDO were significantly elevated in younger ALS vs younger HCs (*p* < 0.05, Figure 3A–C). The sCD27 and sGITR levels demonstrated increases in older ALS patients compared to older HCs, and in younger ALS patients relative to younger HC (*p* < 0.05, Figure 3D–E).

**Table 3.** The results of serum checkpoint detected by luminex.

	ALS (Median [IQR])	HC (Median [IQR])	^†^ *p*	Mimic (Median [IQR])	*p
sGITR	57.33 [42.32–72.92]	28.13 [15.06–40.99]	<0.001	46.11 [26.78–57.33]	0.052
sHVEM	0.00 [0.00–0.36]	0.31 [0.00–0.31]	0.280	0.00 [0.00–0.00]	0.422
sCD27	644.11 [323.65–1416.35]	113.82 [69.12–216.69]	<0.001	266.42 [116.16–371.27]	0.020
sCD28	123.97 [62.01–177.63]	74.29 [46.2–101.38]	0.082	48.65 [17.47–109.06]	0.014
sCD137	93.77 [76.43–122.02]	39.16 [20.95–52.95]	<0.001	66.72 [46.08–95.43]	0.084
sBTLA	951.23 [596.16–1264.94]	250.48 [126.76–583.60]	<0.001	568.23 [314.875–815.75]	0.050
sIDO	33.79 [23.16–52.57]	11.35 [8.18–24.07]	<0.001	22.77 [14.76–39.17]	0.058
sLAG-3	37.49 [28.54–54.10]	19.27 [15.02–28.40]	<0.001	31.71 [25.70–37.85]	0.288
sTIM-3	975.81 [676.13–1302.80]	690.85 [597.46–974.32]	0.124	614.13 [471.30–877.79]	0.040
sPD-1	99.23 [69.89–132.80]	28.80 [19.93–60.92]	<0.001	70.23 [54.91–100.56]	0.050
sPD-L1	1.11 [0.80–1.37]	0.88 [0.78–1.05]	0.104	0.84 [0.73–1.17]	0.198
sPD-L2	2650.08 [2170.79–3265.33]	2053.30 [1244.35–2498.83]	0.004	2220.46 [1817.99–2860.88]	0.306
sCTLA-4	29.02 [22.16–37.95]	19.22 [11.73–25.97]	<0.001	20.24 [18.76–26.11]	0.036
sCD80	112.49 [62.28–171.15]	34.785 [11.75–66.33]	<0.001	58.68 [38.05–118.11]	0.078

Data are expressed as pg/ml.

^†^*p* indicate Bonferroni-corrected *p* values for ALS vs. HC comparisons.

*p indicate Bonferroni-corrected *p* values for ALS vs. mimic comparisons.

ALS, Amyotrophic Lateral Sclerosis; HC, Healthy control; Mimic, patients with ALS mimic syndromes; IQR, interquartile range; HC, Healthy controls; sGITR, serum glucocorticoid-induced tumor necrosis; sHVEM, serum Herpesvirus entry mediator; CD, the cluster of differentiation; sBTLA, serum B and T lymphocyte attenuator; sIDO, serum Indoleamine 2,3-dioxygenase; sLAG-3, serum Lymphocyte activation gene-3; sTIM-3, serum T cell immunoglobulin domain and mucin domain-3; sPD-1, serum Programmed cell death 1; sPD-L1/2, serum Programmed cell death 1 ligand 1/2; sCTLA-4, serum Cytotoxic T lymphocyte associate protein-4.

**Figure 4.**
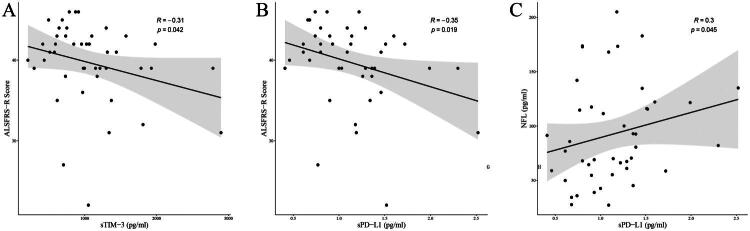
Associations between serum immune checkpoint markers ALSFRS-R scores, and serum neurofilament light chain (NFL) in ALS patients. (A) sTIM3 levels exhibited a negative correlation with ALSFRS-R scores (disease severity). (B,C) sPD-L1 demonstrated dual associations: inversely correlated with ALSFRS-R scores (B) and positively correlated with NFL levels (C).

Subsequently, we analyzed the correlation between serum immune checkpoint markers and the ALSFRS-R score, the monthly decrease in ALSFRS-R, and NFL levels. The sTIM-3 demonstrated inverse correlation with ALSFRS-R score (*p* < 0.05, Figure 4A), while sPD-L1 exhibited dual directional associations: negatively correlated with ALSFRS-R scores and positively associated with NFL (*p* < 0.05, Figure 4B–C). However, these findings did not survive rigorous correction for multiple comparisons (Table S2), and the correlations among the three clinical markers and fourteen immune checkpoint markers are also presented in Table S2. Notably, when dividing the patients into two groups based on the median level of serum immune checkpoint molecules, there were no significant differences in the endpoint events between the two groups (all *p* > 0.05).

## Discussion

Immune checkpoint molecules, including co-inhibitory and co-stimulatory signaling receptors, can regulate T cell proliferation and activity through either stimulation or inhibition. The co-inhibitory molecules primarily include BTLA, IDO, TIM-3, LAG-3, PD-1, PD-L1, PD-L2, CTLA-4/CD152, and CD80. In contrast, the co-stimulatory molecules include GITR, HVEM, CD27, CD28, and CD137/4-1BB [20]. PD-1, as one of the most prevalent immune checkpoint molecules found in T cells, binds to its ligands PD-L1 and PD-L2, thereby inhibiting T cell functions [21]. Our study revealed an elevated proportion of PD-1 in CD4^+^T cells in ALS patients, along with significantly increased PD-1 expression in Th9 and Th17 cells compared to HC. Furthermore, ALS patients with higher PD-1 levels on CD4^+^ T cells exhibited a higher incidence of endpoint events. However, we did not find a correlation between PD1 proportions and ALSFRS-R score, the rate of decline in ALSFRS-R (ALSFRS-R/month), or NFL. This discrepancy may stem from fundamental differences in what these biomarkers represent. Membrane-bound PD-1 reflects local immune modulation, such as T-cell modulation in peripheral blood, whereas ALSFRS-R captures global neurological function [22], and NFL serves as a marker of widespread axonal injury [23]. Their levels may be influenced by multiple factors, including neurodegeneration, glial activation, and other non-immune mechanisms, their relationship with PD-1 may not be direct or linear. Additionally, NFL integrate diverse pathological processes (such as neuroinflammation, blood–brain barrier integrity), which could obscure PD-1–specific effects [23]. Conversely, the role of PD-1 in immune suppression could indirectly affect neurodegeneration without a linear relationship to functional scores. Further research, including PD-1 modulation studies, will be essential to clarify these interactions.

The upregulation of PD-1 may be attributed to the IDO–Kyn–AchR pathway. In current study, it found that serum sIDO levels was increased, while the level of IFN-γ were significantly elevated in ALS, as reported in a previous study [24]. Furthermore, previous research has demonstrated that IFN-γ released by T cells prompts the TCR to upregulate tryptophan transporter proteins and IDO. This process activates AchR through the IDO–Kyn–AchR pathway, which binds to the PD-1 promoter to initiate PD-1 expression [25]. Another study indicated that the PD-1^+^CD4^+^ T cells can increase the transcription of signal transducer and activator of transcription 3 (STAT3), leading to enhanced production of transforming growth factor-β (TGF-β) and IL-17A [26]. Our result also suggested that the PD-1 expression was higher in Th17 cells in ALS patients. Together with prior studies, our findings imply the Th17 cells and their characteristic production IL-17A may contribute to a shifted immune-tolerance status, potentially contributing to the motor neuron (MN) degeneration in ALS [27]. Additionally, Th9 cells exhibiting high PD-1 levels enhance the proliferation of activated CD8^+^T cells [28]. Thus, the expression of PD1 on CD4^+^T cells may influence MN degeneration by modulating cytokine secretion and immunophenotype, thereby participating in the pathology of ALS. Furthermore, our previous study revealed a significant increase in the expression of eomesodermin (EOMES) in CD4^+^T cells in ALS [4]. The upregulated of inhibitory molecules such as BTLA, TIM-3, LAG-3, PD-1, along with change in transcription factors like T-bet and EOMES, are important markers of exhausted T cells [29–31]. Therefore, CD4^+^T cells expressing higher levels of PD-1 and EOMES may represent a subset of exhausted T cells.

The hallmark of exhausted T cells is the heightened expression of inhibitory receptors, including LAG-3, PD-1/PD-L1/2, TIM-3, and CTLA-4 (CD152) [32,33]. These molecules function as immune checkpoint regulators. Hence, we intended to measure the levels of other immune checkpoint molecules to reflect the immune status of ALS patients. We also explored the potential for these molecules to serve as diagnostic biomarkers and therapeutic targets for ALS. However, detecting all these molecules *via* flow cytometry can be challenging, as some require specific stimulation conditions for accurate detection.

Soluble immune checkpoints can be generated through two main mechanisms: Immune cell secretion [20] or membrane-bound checkpoint cleavage [34,35]. These soluble forms serve as functional elements of their membrane-bound counterparts and may act as potential biomarkers for disease diagnosis and treatment development [36,37]. Additionally, they can influence disease progression through positive or negative immune modulation [20]. In this study, we examined the levels of 14 immune checkpoint molecules in serum of ALS patients and Mimics using Luminex technology. Notably, our results indicated that both the expression of inhibitory and stimulatory molecules was altered in ALS patients. Furthermore, we found negative correlations between the ALSFRS-R score and inhibitory molecules such as sTIM-3, and sPD-L1. However, only the inhibitory molecules sPD-L1 showed a correlation with serum NFL levels in ALS patients. One previous study found no difference in sPD-L1 levels between ALS patients and healthy controls [14]. This discrepancy may be attributed to methodological differences: the value of sPD-L1 was relatively low 0.7 to 1.3 pg/ml. Comparing to the prior study, we employed Luminex technology, which offers higher sensitivity and a broader dynamic range. Similar observations have been reported in other studies comparing these two platforms, where Luminex platform demonstrated better performance compared to ELISA [38]. Overall, this study clarifies the relationship between ALS and the various immune checkpoint molecules.

In our study, we observed a significant increase in sPD-1 levels in ALS patients, consistent with findings from a previous study [14]. We also discovered that sPD-L1 levels were associated with the ALSFRS-R score and NFL level. Additionally, we noted alterations in other serum immune checkpoint molecules in ALS patients, encompassing both stimulatory and inhibitory molecules. Notably, sGITR levels were significantly increased in ALS patients, aligning with prior results indicating that higher expression of GITR in SOD1 mice is correlated with shorter survival [39].

BTLA, TIM-3, CTLA-4, IDO, and CD80 are all co-inhibitory molecules. BTLA is primarily expressed on naïve CD4^+^T and CD8^+^T cells in peripheral blood, while TIM-3 serves as a negative regulator of the immune response and is widely expressed across various immune cells, including T cells. CTLA-4 is mainly found on activated T cells, and IDO is primarily expressed in endothelial cells, macrophages, dendritic cells, and fibroblasts. CD80 is predominantly present on activated T cells, B-cells, and monocytes. There may be a correlation between the increased expression of these molecules on cells and elevated levels of their soluble forms [40–42]. Excessive levels of sBTLA, sTIM-3, and sCTLA-4 are likely to diminish cellular immune tolerance through competition [40,43]. Additionally, sIDO may participate in the IDO–Kyn–AChR pathway, which initiates the expression of PD-1, as mentioned earlier. The soluble form of CD80 can inhibit the inhibition effects of the PD-1/PD-L1 pathway, restoring T-cell activation and demonstrating greater efficacy in PD-1/PD-L1 blockade [44]. Conversely, CD27, CD28, and CD137 are co-stimulatory molecules. CD27, a member of the tumor necrosis factor receptor (TNFR) superfamily, is predominantly expressed in T cells but is also found in B and NK cells [45]. The soluble form of CD27 (sCD27) is produced when membrane-bound CD27 is cleaved and is elevated in various diseases and malignancies [46]. For instance, elevated sCD27 levels in the early stages of Huntington’s disease indicate T cell-mediated neuroinflammation [47]. Similarly, sCD28, a variant of CD28 found in peripheral blood, triggers IL-6 secretion upon binding to CD80/CD86 molecules expressed on DC [48]. CD137 is primarily expressed on activated T cells, while its soluble form, sCD137, can antagonize CD137 and regulate the function of effector T cells [49]. Furthermore, recombinant CD137 has been shown to inhibit Th1 and Th2 polarization *in vitro*, which in turn reduced the secretion of IL-13, TNF, and IFN-γ [49]. However, it may also play a negative regulatory role in the immune response [50]. The alterations in the levels of these soluble molecules provide insight into the mechanism underlying elevated serum inflammation in ALS patients.

Some limitations of this study should be acknowledged. Firstly, the origins of the soluble immune checkpoint molecules are not yet fully understood. Second, while we investigated the membrane-bound form of PD-1, we did not elaborate on the expression of membrane-bound forms of the other molecules. Third, in the present study, familial ALS cases were excluded due to a hypothesis that genetic background may influence the disease prognosis and create bias. However, due to resource constraints (insufficient funding for systematic genetic testing) and ethical considerations (participants’ reluctance to undergo optional genetic screening), we were unable to comprehensively assess the genetic status of all participants. Fourth, our analysis focused on only a subset of immune checkpoints, and additional immune checkpoints warrant further exploration. Fifth, while we report exploratory correlations between immunophenotypes and clinical parameters, these analyses did not survive rigorous correction for multiple comparisons. These findings should be considered hypothesis-generating and require validation in larger cohorts. Finally, this study was conducted at a single center with a limited number of cases, and it lacked longitudinal data on immune checkpoints. These findings require validation in multicenter cohorts with larger sample sizes, longitudinal biomarker measurements, and geographically diverse populations. We believe that further investigations into the expression of these immune checkpoint molecules in ALS patients are essential. Such research could explore their potential as biomarkers for ALS and assess whether drugs targeting immune checkpoints may benefit ALS patients.

## Conclusions

In the present study, we demonstrated that both membrane-bound and soluble PD-1 were significantly elevated in ALS patients and correlated with disease progression. Cells expressing high levels of PD-1 may represent a population of exhausted T cells, some of which might regain their function and proliferation capacity following PD-1 blockade therapy [33]. Additionally, we observed alterations in various immune checkpoint molecules in ALS patients, which were associated with a higher incidence of endpoint events. This was assessed through the detection of 14 serum-soluble immune checkpoint molecules, suggesting the presence of an altered immune expression profile in ALS. Overall, this study evaluates a broad spectrum of immune checkpoints in ALS simultaneously. However, the detailed mechanisms by which serum immune checkpoints are related to the immune response in ALS remain to be elucidated.

## Supplementary Material

Supplemental Material

## Data Availability

The authors do not have permission to share data due to Chinese laws. However, anonymized data can be made available to qualified investigators on request from the corresponding authors S Chen and ZY Zou.

## Abbreviations

ALS: Amyotrophic lateral sclerosis
PD-1: programmed death-1
PD-L1/2: programmed death ligands-1/2
SOD1: superoxide dismutase 1
CD: cluster of differentiation
Th cell: Helper T cell
Tfh: Follicular helper T cell
HC: Healthy controls
PN: peripheral neuropathy
CIDP: chronic inflammatory demyelinating polyneuropathy
GBS: Guillain Barre Syndrome
IMNM: immune-mediated necrotizing myopathy
ALSFRS-R: ALS Functional Rating Scale-Revised
CXCR: CXC chemokine receptor
CCR: CC chemokine receptor
NFL: Neurofilament light chain
sGITR: serum glucocorticoid-induced tumor necrosis factor receptor
sHVEM: serum herpesvirus entry mediator
sBTLA: serum B and T lymphocyte attenuator
sIDO: serum Indoleamine-2,3-Dioxygenase
sLAG-3: serum lymphocyte activation gene-3
sTIM-3: serum T-cell immunoglobulin and mucin-domain containing 3
sCTLA-4: serum cytotoxic T lymphocyte-associated antigen-4
SIMOA: single-molecule array
SD: standard deviation
ROC: Receiver operating characteristic curve
AUC: area under the curve
STAT3: signal transducer and activator of transcription 3
IFN-γ: Interferon γ
TGF-β: transforming growth factor-β
MN: motor neurons
EOMES: eomesodermin
