# Supplementary material for: Immune checkpoint changes correlate with the progression and prognosis of amyotrophic lateral sclerosis
Source: Ann Med. 2025 Aug 3;57(1):2540023. doi: 10.1080/07853890.2025.2540023 (PMC12322990; doi:10.1080/07853890.2025.2540023)
Supplement: Supplemental Material [file IANN_A_2540023_SM9799.zip › suppl_data/Figure caption.docx]

**Figure S1 The gating strategies of flow cytometry in this study.**

**Figure S2 Survival analysis by PD-1 expression levels in CD8+ T cells, Th9 cells, and Th17 cells.** Kaplan-Meier survival curves comparing patients with amyotrophic lateral sclerosis (ALS) stratified by high vs. low PD-1 expression (using median expression levels as the threshold) in (A) CD8+ T cells, (B) Th9 cells, and (C) Th17 cells. No significant differences in survival were observed between high and low PD-1 groups for any subset (p > 0.05).

**Figure S3 The levels of serum checkpoint markers tested by Luminex in patients with ALS compared to HC, and specific diseases among Mimics.** (A-L) Panels displayed comparative levels of sPD-1, sBTLA, sCTLA-4, sCD27, sCD28, sTIM-3, sGITR, sCD137, sIDO, sCD80, sLAG-3, and sPD-L2 across ALS, HC and various ALS-mimic diseases.
