# Supplementary material for: Immune checkpoint changes correlate with the progression and prognosis of amyotrophic lateral sclerosis
Source: Ann Med. 2025 Aug 3;57(1):2540023. doi: 10.1080/07853890.2025.2540023 (PMC12322990; doi:10.1080/07853890.2025.2540023)
Supplement: Supplemental Material [file IANN_A_2540023_SM9799.zip › suppl_data/Table S1.docx]

| Target | Clone | Fluorochrome | Vendor |
| --- | --- | --- | --- |
| CD3 | UCHT1 | PerCP.Cy5.5 | BioLegend |
| CD4 | SK3 | FITC | BioLegend |
| CXCR3 | G025H7 | BV785 | BioLegend |
| CCR4 | L291H4 | APC-fire750 | BioLegend |
| CCR6 | G034E3 | BV605 | BioLegend |
| CXCR5 | MU5UBEE | BV421 | eBioscience |
| PD1 | EH12.2H7 | APC | BioLegend |

Table S1 The antibodies used for flow cytometry in this study
