# Supplementary material for: Immune checkpoint changes correlate with the progression and prognosis of amyotrophic lateral sclerosis
Source: Ann Med. 2025 Aug 3;57(1):2540023. doi: 10.1080/07853890.2025.2540023 (PMC12322990; doi:10.1080/07853890.2025.2540023)
Supplement: Supplemental Material [file IANN_A_2540023_SM9799.zip › suppl_data/Table_S2.docx]

Table S2 Correlation among immune checkpoint markers and ALSFRS-R score, ALSFRS-R progression rate and NFL levels.

|  | ALSFRS-R score | | | ALSFRS-R progression rate | | | NFL levels | | |
| --- | --- | --- | --- | --- | --- | --- | --- | --- | --- |
|  | R-value | p-value | Adjust p-value | R-value | p-value | Adjust p-value | R-value | p-value | Adjust p-value |
| sGITR | -0.459 | 0.002 | 0.068 | 0.101 | 0.513 | 0.615 | 0.155 | 0.315 | 0.473 |
| sHVEM | -0.312 | 0.039 | 0.170 | 0.151 | 0.329 | 0.477 | 0.163 | 0.290 | 0.451 |
| sCD27 | -0.233 | 0.128 | 0.336 | -0.030 | 0.848 | 0.869 | 0.198 | 0.197 | 0.363 |
| sCD28 | -0.198 | 0.199 | 0.363 | -0.065 | 0.675 | 0.766 | 0.216 | 0.159 | 0.347 |
| sCD137 | -0.359 | 0.017 | 0.111 | 0.272 | 0.074 | 0.223 | 0.198 | 0.199 | 0.363 |
| sBTLA | -0.417 | 0.005 | 0.068 | 0.124 | 0.424 | 0.539 | 0.426 | 0.004 | 0.068 |
| sIDO | -0.213 | 0.165 | 0.347 | -0.036 | 0.818 | 0.859 | 0.179 | 0.246 | 0.431 |
| sLAG-3 | -0.136 | 0.379 | 0.513 | -0.037 | 0.814 | 0.859 | 0.166 | 0.280 | 0.451 |
| sTIM-3 | -0.308 | 0.042 | 0.170 | 0.047 | 0.762 | 0.842 | 0.255 | 0.095 | 0.265 |
| sPD-1 | -0.392 | 0.009 | 0.090 | 0.131 | 0.397 | 0.521 | 0.336 | 0.026 | 0.134 |
| sPD-L1 | -0.354 | 0.019 | 0.111 | 0.144 | 0.352 | 0.492 | 0.304 | 0.045 | 0.170 |
| sPD-L2 | -0.173 | 0.262 | 0.439 | 0.014 | 0.930 | 0.930 | 0.298 | 0.049 | 0.170 |
| sCTLA-4 | -0.226 | 0.141 | 0.347 | 0.066 | 0.671 | 0.766 | 0.294 | 0.053 | 0.170 |
| sCD80 | -0.363 | 0.015 | 0.111 | 0.109 | 0.480 | 0.592 | 0.218 | 0.155 | 0.347 |

ALS, Amyotrophic Lateral Sclerosis; ALSFRS-R, Amyotrophic Lateral Sclerosis; ALSFRS-R progression rate, calculated by the standard ALSFRS-R progression rate formula: Progression Rate = (48 − ALSFRS-R Score)/Disease Duration (months); adjust p-value, Benjamini-Hochberg FDR-adjusted p-values.
