# Supplementary figures and images for: Immune checkpoint changes correlate with the progression and prognosis of amyotrophic lateral sclerosis
Source: Ann Med. 2025 Aug 3;57(1):2540023. doi: 10.1080/07853890.2025.2540023 (PMC12322990; doi:10.1080/07853890.2025.2540023)

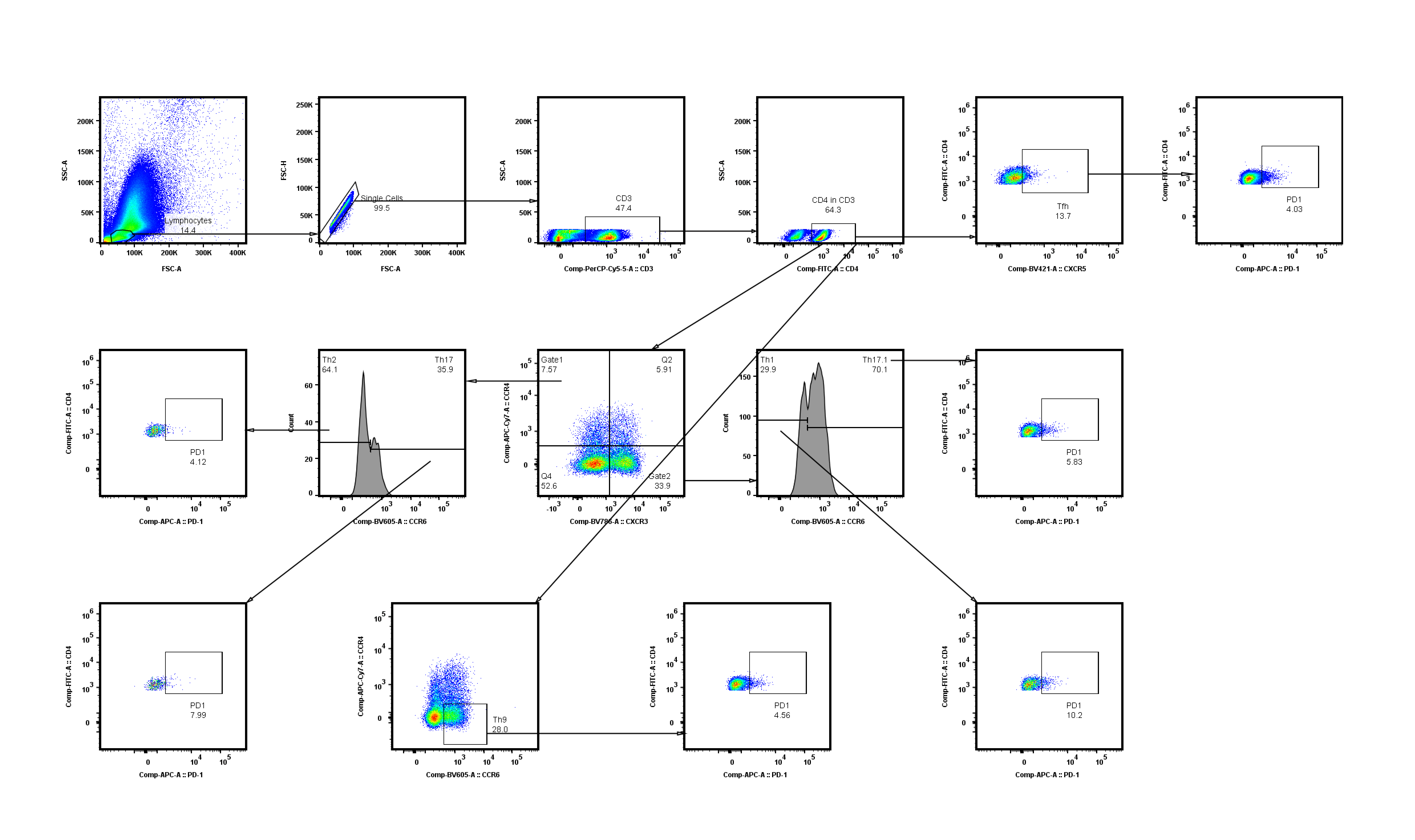

Supplement: Supplemental Material [file IANN_A_2540023_SM9799.zip › suppl_data/Figure S1.tif]

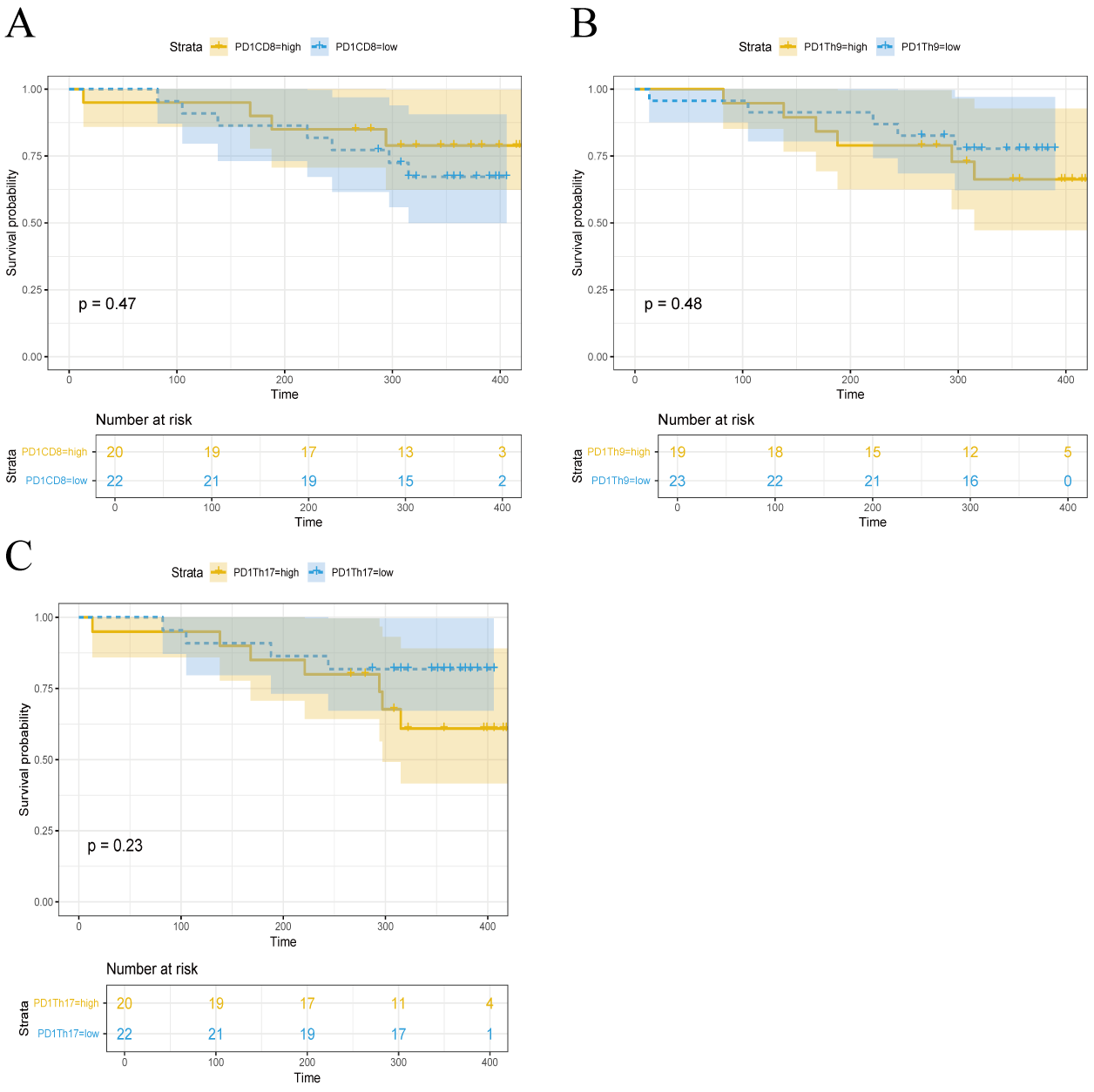

Supplement: Supplemental Material [file IANN_A_2540023_SM9799.zip › suppl_data/Figure S2.tif]

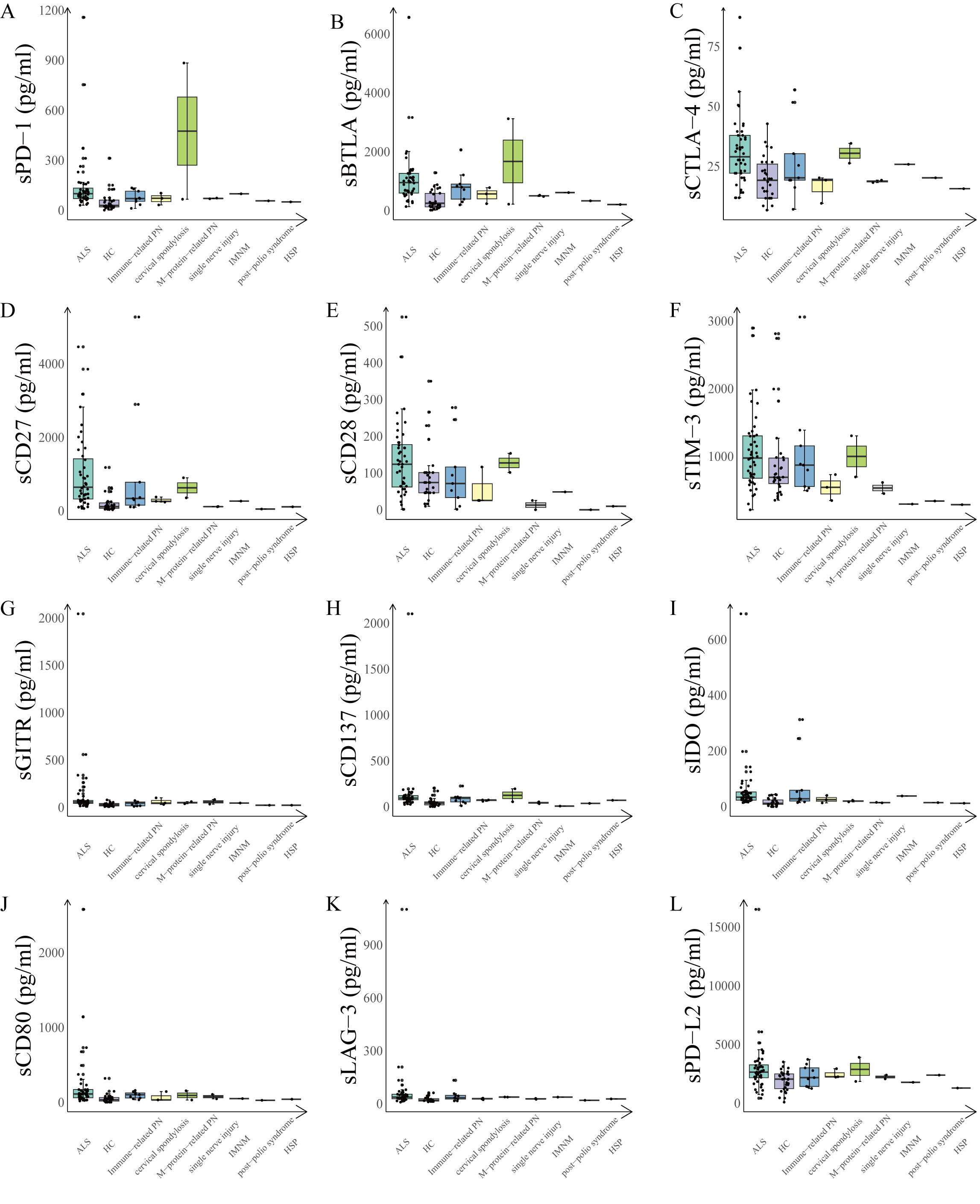

Supplement: Supplemental Material [file IANN_A_2540023_SM9799.zip › suppl_data/Figure S3.tif]
